# Cognitive integrity in Non‐Demented Individuals with Alzheimer’s Disease Neuropathology is associated with preservation and remodeling of dendritic spines

**DOI:** 10.1002/alz.087096

**Published:** 2025-01-03

**Authors:** Jutatip Guptarak, Pietro Scaduto, Batbayar Tumurbaatar, Daniel Jupiter, Giulio Taglialatela, Anna Fracassi

**Affiliations:** ^1^ University of Texas Medical Branch, Galveston, TX USA

## Abstract

**Background:**

Alzheimer’s disease (AD) is the most prevalent neurodegenerative disorder leading to dementia. The existence of individuals who remain cognitively intact despite presenting histopathological signs of AD, here referred to as “Non‐demented with AD neuropathology” (NDAN), suggests that some mechanisms are triggered to resist cognitive impairment. These individuals are distinguished by the presence of highly phagocytic microglia capable of clearing damaged synapses near plaques, mitigating further damage to axons and dendrites.

**Method:**

We conducted a comparative analysis of dendritic spines morphology in the *post‐mortem* frontal cortex of NDAN individuals, AD patients, and age‐matched healthy controls. Our investigation included an in‐depth examination of synaptic structures both near and far from Aβ plaques, quantifying aspects such as dendrite length, diameter, spine density, and types. We expanded our research to investigate levels and distribution of Pin1, identified as a potential key player in the protective mechanisms against AD, influencing the regulation of dendritic spine formation and maintenance.

**Result:**

Within 100 µm of Aβ plaques, significant synaptic toxicity was observed in all groups. However, in areas distal to plaques, NDAN exhibited significantly higher spine density than AD, suggesting the existence of a compensatory mechanism. We also measured the relative abundance of four spine types: mushroom, stubby, filopodia, and long thin, finding stubby spines to be the most common across all groups. Mushroom spines, the least dynamic, were significantly more abundant in AD compared to NDAN and control subjects. Conversely, NDAN individuals showed a higher density of more dynamic and plastic spines, such as filopodia and long thin spines, than AD. These findings suggest that the rearrangement of dynamic dendritic spines in NDAN may underlie the ability of these individuals to replace damaged synapses and preserve cognitive integrity. Furthermore, our results revealed lower expression of Pin1 in AD patients than control and NDAN groups, across regions, proximal and distal to plaques. This finding suggests that reduced Pin1 expression in AD may contribute to the compromised synaptic integrity and plasticity observed in these individuals.

**Conclusion:**

This study sheds light on the potential mechanisms allowing NDAN individuals to retain cognitive function despite AD pathology, offering insights for future therapeutic strategies.

